# Integrated single-cell and bulk transcriptomic analysis reveals shared pathogenesis and prognostic biomarkers in breast and thyroid cancers

**DOI:** 10.3389/fimmu.2025.1710915

**Published:** 2026-01-12

**Authors:** Bingbing Shen, Jiayi Jiang, Xinyue Zhang, Cheng Yi, Jiaye Liu, Zhihui Li, Yu Ma

**Affiliations:** 1 Division of Thyroid Surgery, Department of General Surgery, Laboratory of Thyroid and Parathyroid Diseases, Frontiers Science Center for Disease-Related Molecular Network, West China Hospital, Sichuan University, Chengdu, China; 2 Department of Respiratory and Critical Care Medicine, Frontiers Science Center for Disease-related Molecular Network, Center of Precision Medicine, Precision Medicine Key Laboratory of Sichuan Province, West China Hospital, Sichuan University, Chengdu, China; 3 Department of Ophthalmology, Second Affiliated Hospital of Xi′an Jiaotong University, Xi′an, China; 4 Department of Nuclear Medicine, West China Hospital, Sichuan University, Chengdu, China; 5 Frontiers Medical Center, Tianfu Jincheng Laboratory, Sichuan University, Chengdu, China

**Keywords:** breast cancer, co-pathogenesis, single-cell transcriptome, SMR3B, thyroid cancer

## Abstract

**Background:**

Breast cancer (BC) and thyroid cancer (TC) are two hormonally regulated malignancies with increasing evidence of significant comorbidity. However, the underlying molecular mechanisms contributing to their co-occurrence remain unclear.

**Purpose:**

This study aimed to elucidate the shared pathogenesis of BC and TC and to identify common prognostic biomarkers and therapeutic targets.

**Study design:**

An integrative bioinformatics study combining single-cell and bulk RNA sequencing data was conducted to investigate shared molecular features between BC and TC.

**Methods:**

Differentially expressed genes (DEGs) were identified and subjected to functional enrichment analysis. Single-cell transcriptome analysis was performed to characterize tumor microenvironment composition and malignant cell heterogeneity. Copy number variation (CNV) and non-negative matrix factorization (NMF) analyses were used to identify key gene expression modules. Weighted gene co-expression network analysis (WGCNA) was applied to bulk transcriptomic data to determine critical cell populations. A prognostic signature was constructed using 101 machine learning algorithms, and functional assays were conducted to validate gene function.

**Results:**

Enrichment analyses indicated that the JAK-STAT signaling pathway and cytokine–cytokine receptor interaction are shared pathogenic mechanisms. Single-cell analysis revealed immune cell involvement and malignant cell heterogeneity. Modules MP2, MP4, and MP5 were identified as critical in both cancers. WGCNA highlighted SFRP2+ fibroblasts and HLA_DPB1+ myeloid cells as key players in tumorigenesis. A prognostic model was developed, and SMR3B was validated as a shared prognostic gene that influenced proliferation, migration, and invasion in both BC and TC.

**Conclusion:**

This study provides comprehensive insights into the shared molecular mechanisms of BC and TC and identifies SMR3B as a promising prognostic biomarker and therapeutic target, offering new avenues for managing patients at dual risk.

## Background

Breast cancer (BC) and thyroid cancer (TC) are among the most prevalent malignancies worldwide (1, 2). According to the 2022 World Cancer Data, BC holds the highest incidence among female cancers, while TC ranks seventh among all cancers globally (2). Growing epidemiological evidence suggests a significant association between these two malignancies. The potential pathogenic link between BC and TC was first proposed by Ron and colleagues in 1984, who hypothesized shared etiological factors (3). Subsequent study has reported a higher incidence of BC in patients diagnosed with TC (4), with Bakos and colleagues identifying 112 genes showing significantly elevated polygenic risk burdens in breast-thyroid cancer patients compared to breast cancer-only cases, supporting a genetic predisposition (5). This bidirectional association has been further substantiated by work from Joseph and Nielsen’s research groups, showing elevated risks of developing BC as a second primary malignancy following TC diagnosis, and vice versa (6, 7). Multiple mechanisms may underlie this comorbidity, including (1): shared genetic susceptibility loci (2), complex hormonal interactions (particularly involving estrogen and thyroid hormone pathways) (3), overlapping oncogenic signaling cascades (e.g., PI3K/AKT) (4), environmental exposures, and (5) iatrogenic effects of prior cancer therapies (8–10). Despite these insights, the precise mechanisms driving the co-occurrence of BC and TC remain elusive. Therefore, an in-depth investigation into their common pathogenesis and molecular underpinnings is warranted and may yield valuable implications for prevention and management strategies.

Recent advances in single-cell sequencing technologies have revolutionized our ability to dissect cellular heterogeneity and functional diversity in both physiological and pathological contexts (11–13).

**Figure 1:**
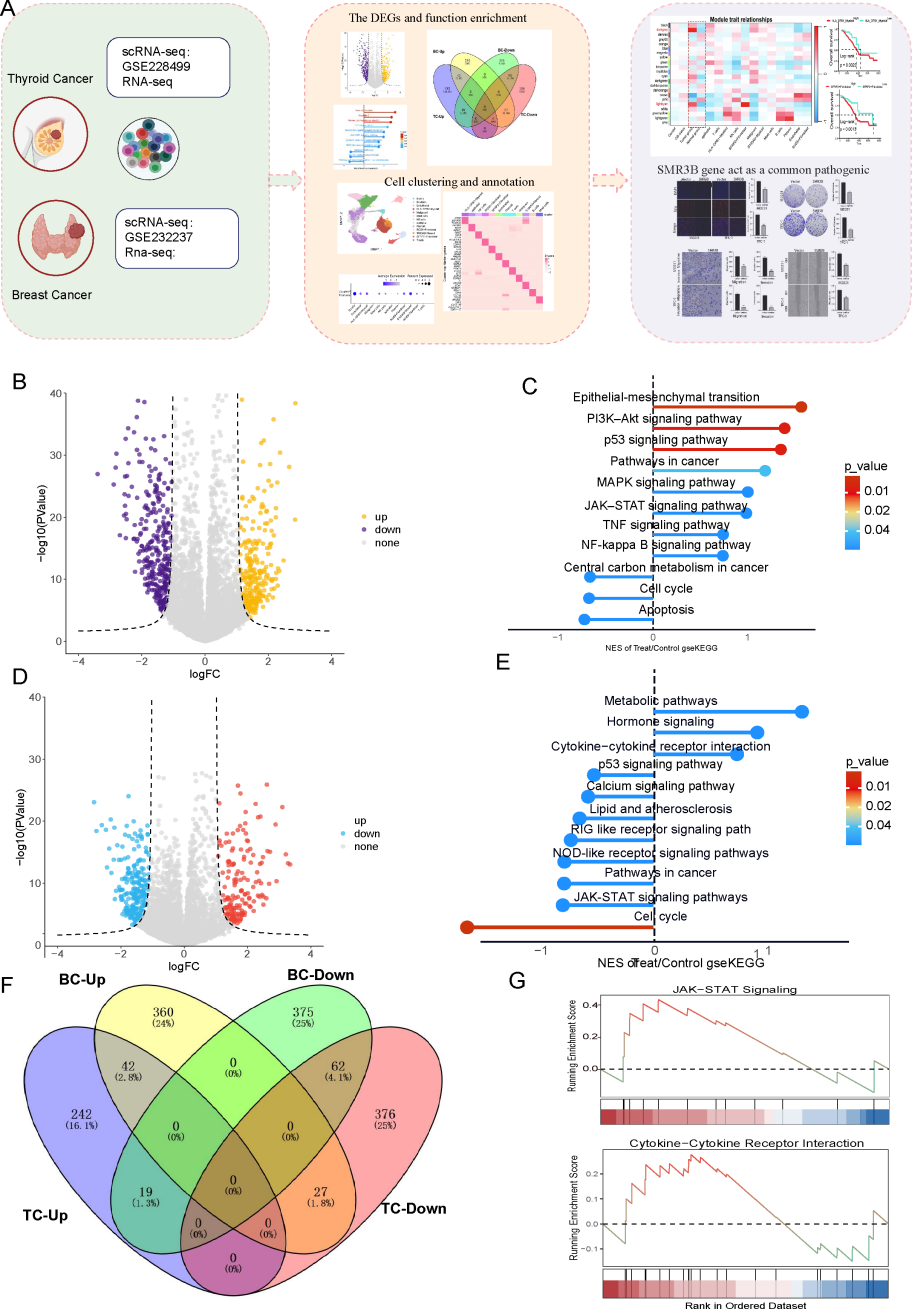
Differential expression and function enrichment analysis in BC and TC. **(A)** The study flowchart displayed analysis steps. **(B)** Identification of DEGs in BC. **(C)** KEGG analysis of DEGs in BC. **(D)** Identification of DEGs in TC. **(E)** KEGG analysis of DEGs in TC. **(F)** VENN plot identified upregulated and downregulated genes. **(G)** GSEA showed function enrichment of common DEGs.

These approaches have significantly advanced precision medicine by enabling detailed characterization of tumor microenvironments, immunotherapy responses, and tissue regeneration mechanisms. In the current study, we leverage BC single-cell transcriptomic data (14) and TC data (15), integrated with bulk RNA-seq datasets, to perform a comprehensive analysis of co-pathogenesis. **Figure 1A** shows the study flowchart.

## Methods

### Data collection and processing

Single-cell datasets GSE228499 (BC) (14) and GSE232237 (TC) (15) were retrieved from GEO (https://www.ncbi.nlm.nih.gov/geo/) and preprocessed via Seurat for quality control, ensuring robust transcriptomic analysis. We used Harmony for batch correction across multiple scRNA-seq datasets. Key parameters included theta = 2, lambda = 1, and nclust = 50. Subsequently, the raw matrix underwent quality control: those cells with <200 genes or >15% mitochondrial genes were excluded. Sequential steps included identifying 2000 highly variable genes (Seurat’s FindVariableFeatures), PCA-based dimensionality reduction (RunPCA), and cell clustering via FindNeighbors and FindClusters algorithms. To investigate transcriptional differences in BC and TC, we integrated three BC bulk RNA-seq datasets, included GSE21653 (16), GSE88770 (17), TCGA-BRCA and TCGA-THCA cohorts (https://xenabrowser.net/datapages/). To define the 12 major cell types, we first conducted an exploratory analysis of the DEGs in each cluster. Marker genes were identified using the FindAllMarkers function, which applies Bonferroni correction (p.adjust(method = “bonferroni”)) to compute the FDR (i.e., p_val_adj).

### Identification of differentially expressed genes, gene set enrichment, and functional annotation

DEGs were identified using the LIMMA package, applying thresholds of P < 0.05 and |log_2_ fold change| > 1. the Benjamini–Hochberg (BH) false discovery rate (FDR) method, with FDR < 0.05 considered statistically significant. Functional enrichment analysis of the DEGs was conducted using the “ClusterProfiler” R package, incorporating Gene Ontology (GO) and Kyoto Encyclopedia of Genes and Genomes (KEGG) annotations. The GO analysis encompassed biological processes, cellular components, and molecular functions. Additionally, gene set enrichment analysis (GSEA) was performed to explore pathway enrichment across different molecular subtypes based on gene sets obtained from the Molecular Signatures Database (MSigDB, https://www.gsea-msigdb.org/gsea/msigdb).

### Construction of machine learning models

To construct a reliable and high-performing prognostic system for BC and TC, we established a comprehensive machine learning framework that systematically evaluated ten core algorithms and their 101 pairwise integrations. This ensemble approach encompassed models such as Random Survival Forest (RSF), Elastic Net variants (including Lasso and Ridge), Stepwise Cox regression, CoxBoost, Partial Least Squares for Cox models (plsRcox), Supervised Principal Component Analysis (SuperPC), Generalized Boosted Regression Modeling (GBM), and Survival Support Vector Machines (Survival-SVM). The workflow comprised four main phases: (a) Initial screening of survival-related genes (SRGs) in the TARGET cohort using univariate Cox regression; (b) Input of selected genes into all 101 algorithm combinations, with model training and tuning guided by leave-one-out cross-validation (LOOCV); (c) Rigorous external validation of the resulting models across four independent datasets—GSE21653, GSE88770, TCGA-BRCA, and TCGA-THCA; (d) Final model selection based on the average Harrell’s concordance index (C-index) across validation cohorts, prioritizing both accuracy and generalizability. This integrative strategy ensured a balanced evaluation of individual and combined algorithmic strengths, ultimately leading to a robust prognostic signature.

### Clustering, dimensionality reduction, and cell type annotation of scRNA-seq data

Single-cell RNA sequencing (scRNA-seq) data were processed and analyzed using the “Seurat” R package. Data integration across samples was performed using the Harmony algorithm (18–20). Log-normalization was applied to the merged dataset, and the top 2,000 highly variable genes were selected using the FindVariableFeatures function with the “vst” method. Subsequently, gene expression values were scaled using ScaleData, and principal component analysis (PCA) was conducted via RunPCA based on the variable genes. The first 20 principal components were utilized for clustering using FindNeighbors and FindClusters (resolution = 0.6). For dimensionality reduction and visualization, UMAP was applied to the top 30 principal components. Cluster-specific marker genes were identified using the FindAllMarkers function, with criteria set as log fold change > 0.5, minimum percentage expression (min.pct) > 0.25, and adjusted p value < 0.05.

### Estimation of cellular stemness using the CytoTRACE algorithm

Lineage differentiation is generally associated with decreased chromatin accessibility, which can be indirectly assessed through single-cell gene counts. Gulati et al. demonstrated that higher gene counts correlate with a less differentiated, stem-like state (21). Leveraging this observation, they developed CytoTRACE, a computational framework that infers cellular stemness from scRNA-seq data by identifying genes most positively correlated with gene counts. The algorithm assigns each cell a stemness score, reflecting its differentiation status. CytoTRACE has been validated across multiple large-scale datasets and has shown superior performance compared to previous approaches.

### Identification of malignant program of epithelial cells

The CopyKAT algorithm in R was applied to infer genome-wide copy number variations (CNVs) at single-cell resolution, stratifying cells into diploid or aneuploid categories. Subsequent cluster reanalysis leveraged CNV profiles to systematically discriminate malignant cells from non-malignant counterparts (22). Non-negative matrix factorization (NMF) is effective for extracting interpretable features from high-dimensional, non-negative data. In scRNA-seq analysis, it reduces complex gene expression matrices into a few gene programs, aiding the discovery of recurrent cancer-associated patterns (23, 24). GeneNMF, an R package designed for gene module identification, was applied directly to Seurat objects. The analysis was performed using default settings unless otherwise specified, with non-negative matrix factorization implemented via the RcppML (25) backend for efficient and scalable computation. The number of gene modules was determined based on the recommended GeneNMF workflow. Single-cell CNV was inferred using CopyKAT (v1.1.1) (22)based on filtered scRNA-seq count matrices. Gene expression values were normalized against putative diploid reference cells, and large-scale CNVs were called using a sliding window covering five consecutive genes along each chromosome. Genomic segments were classified as amplified (z > 0.1) or deleted (z < −0.1) using standardized deviation thresholds selected to reliably separate aneuploid tumor cells from diploid normal cells, while reducing false positives from transcriptional noise. Tumor and normal cell identities were determined through unsupervised k-medoids clustering applied to CNV profiles. These classifications were subsequently used in downstream analyses, such as differential expression and integration with spatial transcriptomics data.

### Pseudo-time and trajectory analysis

The Slingshot R package was employed to infer cell trajectory trees and pseudo-temporal ordering via diffusion map projections, with myofibroblasts, fibroblasts, and pericytes designated as input cell populations (26). Myofibroblasts were designated as terminal clusters, guided by diffusion map projections and biological rationale derived from prior knowledge (26).

### Weighted gene coexpression network analysis for coexpression network construction

WGCNA was implemented to delineate coexpressed modules, investigate phenotypic associations, and pinpoint intramodular hub genes. The WGCNA-R package was employed to construct genome-wide co-expression networks, prioritizing the top 10,000 highest-variance genes for subsequent functional analyses (27). Network connectivity was assessed by transforming the weighted adjacency matrix into a topological overlap matrix (TOM). Hierarchical clustering generated a dendrogram, where branches demarcated distinct gene modules, with color-coding to differentiate module identities. Genes were algorithmically partitioned into modules based on expression pattern coherence, consolidating functionally analogous genes. Final classification of tens of thousands of genes into discrete modules was achieved through weighted correlation coefficient thresholds.

### Cell type deconvolution via CIBERSORTx

Cellular composition of bulk RNA-seq samples was deconvolved using CIBERSORTx, leveraging a preprocessed single-cell transcriptomic reference dataset (28). Low-quality cells were filtered, expression values were log-normalized, and cell type-specific signature matrices were generated to resolve population-level heterogeneity. CIBERSORTx (https://cibersortx.stanford.edu/) was implemented in high-resolution mode (1,000 permutations) with batch effect correction to resolve cell-type fractions across bulk RNA-seq samples. Validation included Wilcoxon rank-sum tests evaluating cell-type proportion disparities between experimental cohorts.

### Survival analysis and predictive Kaplan–Meier curves

Target datasets (external/internal validation cohorts) were stratified into high-/low-risk groups using the median IRS score. Patients with risk scores above the median were classified as the high-risk group, and those below the median as the low-risk group. Kaplan-Meier survival analysis (survminer R package) assessed intergroup survival disparities, with statistical significance determined by log-rank test (p<0.05). The timeROC package was employed to conduct ROC curve analysis, evaluating the IRS’s prognostic sensitivity/specificity for survival prediction. Model generalizability was validated using TCGA cohorts, with log-rank tests (p<0.05) confirming significant survival disparities (OS, DSS, DFI, PFI) between risk groups.

### Cell–cell communication analysis

The CellCall R package (v1.16.0) was employed to systematically map intercellular communication dynamics and infer intracellular signaling pathways through ligand-receptor interaction profiling across defined cell populations.

### Cell lines experiments

For SMR3B plasmid transfection, TPC-1, K1, MCF7 and MD231 cell lines were transfected with using Lipofectamine 3000 (Invitrogen) at a mass ratio under serum-free conditions. Post-transfection medium replacement occurred at 6 h, followed by functional validation assays (proliferation, migration, invasion) at 48 h post-transfection.

The qPCR of SMR3B expression was performed as follows: Total RNA isolated via TRIzol reagent (Invitrogen) underwent reverse transcription using PrimeScript RT Master Mix (Takara). Amplification reactions were executed on a QuantStudio 5 Real-Time PCR System (Applied Biosystems) with SYBR Green Premix (Roche), employing the following primers: SMR3B forward 5’-TGACTTGGATCTTGGGCCTTT-3’, reverse 5’-GGGCCTCTTTGACTCTCACC-3’; GAPDH forward 5’-CTGGGCTACACTGAGCACC-3’, reverse 5’-AAGTGGTCGTTGAGGGCAATG-3’. Relative expression levels were normalized to GAPDH and quantified using the ΔΔCt method (29).

EdU proliferation assays were conducted by incubating cells with 10 μM EdU reagent (Elabscience, E-CK-A351) for 2 h at 37 °C. Cells were fixed in 4% paraformaldehyde (PFA) for 15 min, permeabilized with 0.5% Triton X-100 for 10 min, and subjected to Click-iT reactions using Alexa Fluor 594 azide. Nuclei were counterstained with DAPI prior to fluorescence imaging.

Colony formation assays were performed by seeding single-cell suspensions (1000 cells/well) into 6-well plates, followed by 14-day culture under standard conditions (37 °C, 5% CO_2_). Colonies were fixed with ice-cold methanol for 15 min, stained with 0.1% crystal violet for 30 min, and quantified via automated threshold analysis in ImageJ.

Wound healing assays were performed by creating uniform linear scratches in confluent cell monolayers using sterile 200 μL pipette tips. Gap closure dynamics were longitudinally assessed at 0/48 hours post-wounding via live-cell imaging (Olympus IX83 inverted microscope, 4× objective). Migration rates were quantified as percentage closure relative to baseline wound area using ImageJ with threshold-based analysis.

### Statistical analysis

The R language was used for all statistical studies (version 4.5.0) and GraphPad Prism (v10.1.0). For the correlation analysis, Spearman’s correlation was used. To examine the differences between these two risk groups, the Wilcoxon test was applied. Statistical significance was defined as *p* value of <0.05. ****p < 0.0001, ***p < 0.001, **p < 0.01, *p < 0.05, ns (not significant), with asterisks annotating significance levels in figures.

## Results

### Differential expression and function enrichment analysis

To explore the comorbid mechanisms of BC and TC, we conducted an initial differential gene expression analysis across the TCGA-BRCA and TCGA-THCA cohorts, followed by a focused identification of DEGs in the TCGA-BRCA transcriptomic data using stringent criteria (|log2FC| ≥1, adjusted *p* < 0.05) to reveal potential co-pathogenic pathways. This revealed 429 significantly up-regulated and 456 down-regulated genes in BC (**Figure 1B**). Subsequent KEGG enrichment analysis of these DEGs highlighted key carcinogenic pathways, including the JAK-STAT signaling pathway, cytokine-cytokine receptor interaction, NOD-like receptor signaling pathway, and metabolic pathways (**Figure 1C**). Transcriptomic analysis of the TCGA-THCA cohort, applying identical thresholds (|log2FC| ≥1, adjusted *p* < 0.05), identified 303 up-regulated and 465 down-regulated genes (**Figure 1D**). KEGG enrichment analysis of these DEGs revealed prominent pathways including metabolic pathways, neuroactive ligand-receptor interaction, cytokine-cytokine receptor interaction, JAK-STAT signaling, and NOD-like receptor signaling (**Figure 1E**). VENN plot shows upregulated and downregulated genes of BC and TC (**Figure 1F**). Enrichment analysis of common DEGs showed significant association with the JAK-STAT signaling pathway and the cytokine–cytokine receptor interaction pathway (**Figure 1G**), indicating that these pathways may serve as a key molecular basis for BC–TC comorbidity.

### A single-cell atlas reveals divergent TME dynamics in BC and TC tissues

**Figure 2:**
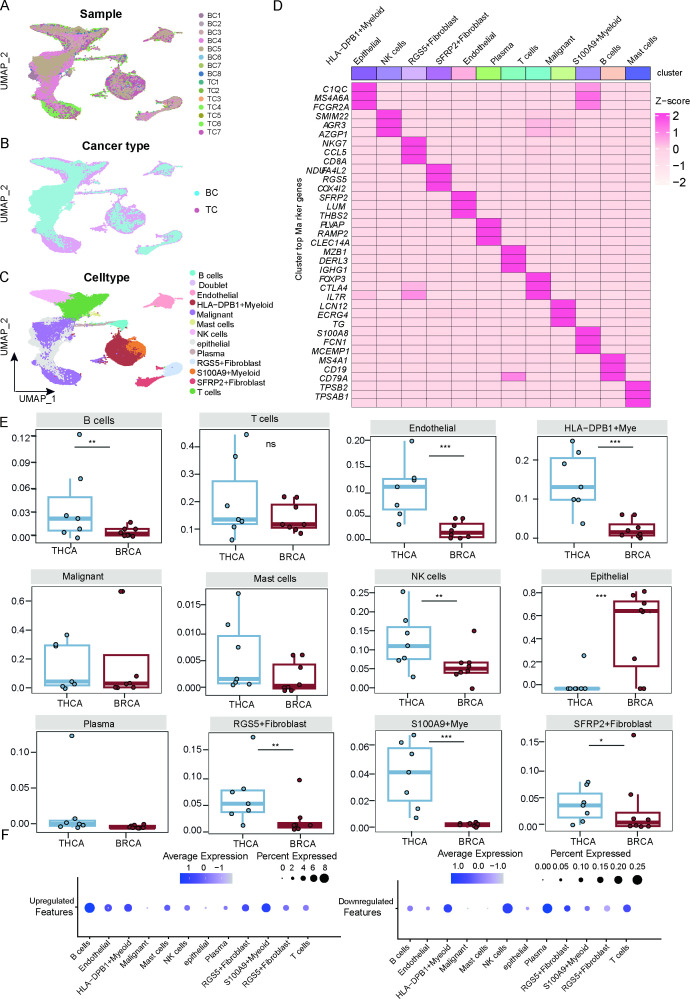
A single-cell atlas reveals divergent TME dynamics in BC and TC tissues. ***p < 0.001, **p < 0.01, *p < 0.05, ns (not significant). **(A)** UMAP plot showing sample types. **(B)** UMAP plot showing tumor types. **(C)** UMAP plot showing cell types. **(D)** Heatmap displaying the top three highly expressed genes for each cell type. **(E)** Boxplots showing the cellular fractions of B cells, T cells, Endothelial, HLA-DBP1+Mye, Malignant, Mast cells, NK cells, epithelial, Plasma, RGS5+Fibroblast, S100A9+Mye and SFRP2+Fibroblast in BC and TC patients. Statistical analysis was performed via one-sided unpaired Wilcoxon tests, with false discovery rate (FDR) correction. **(F)** The upregulated and downregulated DEGs enriched cell types.

To comprehensively analyze the similarities and differences in the tumor microenvironment (TME) between the two cancer types, we integrated single-cell transcriptomic data from 8 BC and 7 TC samples. Following cross-cohort data integration using Harmony method for batch correction, we profiled 60,457 high-quality single cells (BC: 21,923; TC: 38,534), with UMAP visualization confirming distinct cellular distributions (**Figures 2A, B**). Single-cell clustering was performed via the Seurat method with UMAP-based dimensionality reduction. Initial analysis identified 13 cell clusters, from which doublets were computationally excluded, yielding 12 biologically distinct populations: HLA-DPB1+Myeloid (*C1QC*, *MS4A6A*, *FCGR2A*), epithelial (*SMIM22*, *AGR3*, *AZGP1*), NK cells (*NKG7*, *CCL5*, *CD8A*), RGS5+Fibroblasts (*RGS5*, *COX4I2*, *NDUFA4L2*), SFRP2+Fibroblasts (*SFRP2*, *LUM*, *THBS2*), endothelial (*PLVAP*, *RAMP2*, *CLEC14A*), plasma (*MZB1*, *DERL3*, *IGHG1*), T cells (*IL7R*, *FOXP3*, *CTLA4*), malignant (*LCN2*, *ECRG4*, *TG*), S100A9+Myeloid (*S100A8*, *FCN1*, *MCEMP1*), B cells (*MS4A1*, *CD19*, *CD79A*), and mast cells (*TPSB2*, *TPSAB1*, *CPA3*, **Figure 2C-D**). Comparative immune cell analysis revealed distinct enrichment patterns between BC and TC. In TC, significant enrichment was observed for B cells, endothelial cells, HLA-DPB1+Myeloid cells, NK cells, RGS5+Fibroblasts, S100A9+Myeloid cells, and SFRP2+Fibroblasts, whereas epithelial cells were predominantly enriched in BC (**Figure 2E**). Integrated bulk transcriptomic analysis further demonstrated that upregulated genes were primarily localized to B cells, HLA-DPB1+Myeloid, and S100A9+Myeloid populations, while downregulated genes clustered in HLA-DPB1+Myeloid, NK cells, and plasma cells (**Figure 2F**). These findings suggest that immune regulatory mechanisms play distinct roles in TME of the two cancer types.

### Single-cell transcriptomic profiling uncovers malignant heterogeneity and shared functional modules in BC and TC

**Figure 3:**
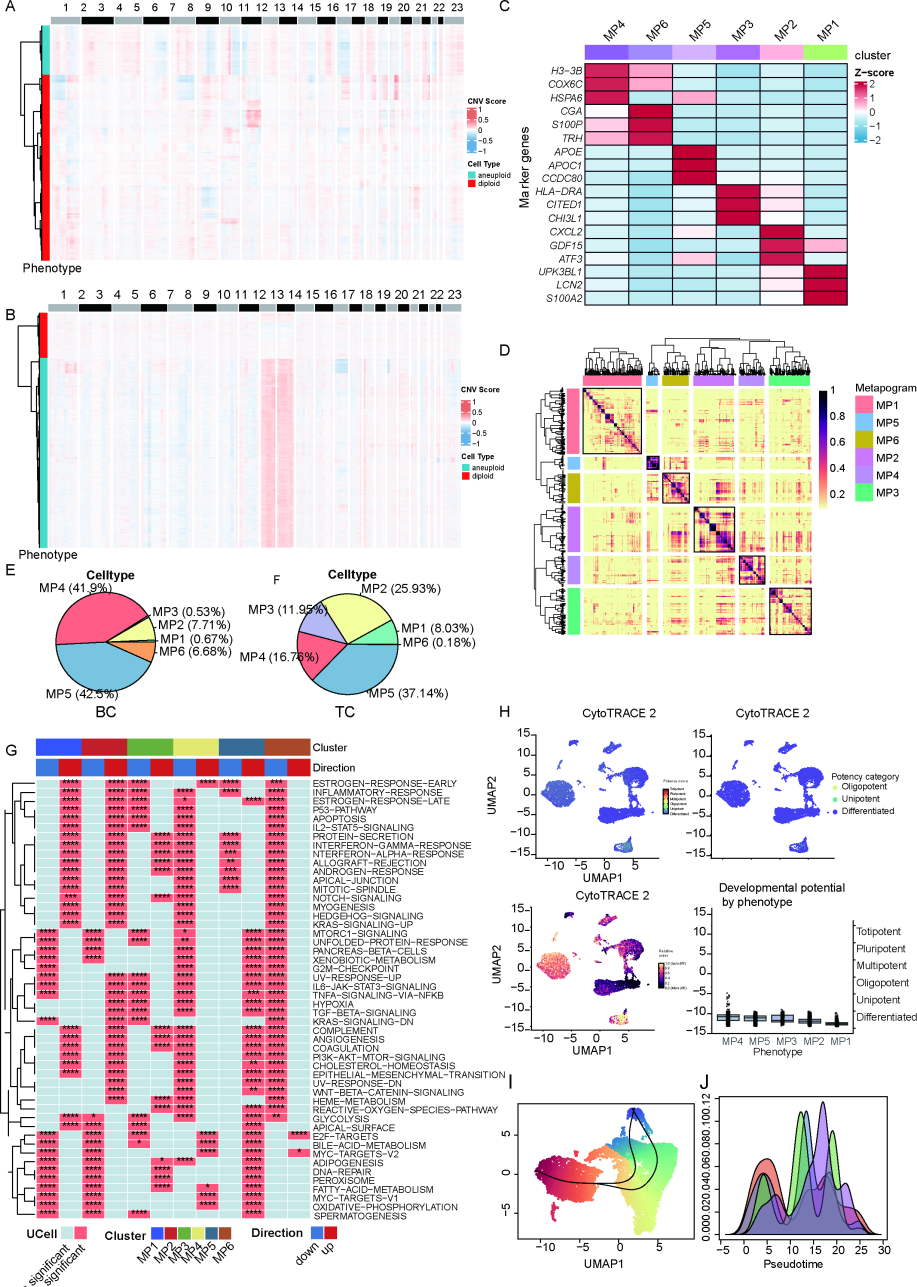
Single-cell transcriptomic profiling uncovers malignant heterogeneity and shared functional modules in BC and TC. ****p < 0.0001, ***p < 0.001, *p < 0.05. **(A)** Inference of aneuploid tumor cells in BC samples via the CopyKAT algorithm. **(B)** Inference of aneuploid tumor cells in TC samples via the CopyKAT algorithm. **(C)** Heatmap depicting displaying the top three highly expressed genes for each correlated Modules based on cNMF algorithm. **(D)** Heatmap depicting displaying six highly correlated Modules. **(E)** The ratio of six highly correlated Modules in BC. **(F)** The ratio of six highly correlated Modules in TC. **(G)** six highly correlated Modules enriched pathways. **(H)** The tumor stemness characteristics using the CytoTRACE algorithm. **(I)** The cell trajectory tree inference and pseudo-time cell ordering inference. **(J)** Cells inferred to have high stemness scores by CytoTRACE analysis.

To characterize the functional heterogeneity of malignant cells, we employed CopyKAT to infer chromosomal aneuploidy in BC and TC cells. Single-cell transcriptomic profiling of malignant epithelial cells revealed distinct ploidy distributions: BC exhibited 4,162 aneuploid versus 11,241 diploid cells (**Figure 3A**), while TC demonstrated 7,331 aneuploid compared to 1,721 diploid cells (**Figure 3B**), highlighting divergent chromosomal instability patterns between these hormone-dependent cancers. To delineate functional heterogeneity within malignant cells, we implemented NMF to resolve gene co-expression modules. This framework prioritizes module-level analysis over raw expression matrices, thereby minimizing cross-sample technical variability and batch effects. Six biologically validated co-expression modules were identified (**Figures 3C, D**): MP4 *(H3-3B*, *COX6C*, *HSPA6*), MP6 (*CGA*, *S100P*, *TRH*), MP5 (*APOE*, *APOC1*, *CCDC80*), MP3 (*HLA-DRA*, *CITED1*, *CHI3L1*), MP2 (*CXCL2*, *GDF15*, *ATF3*), MP1 (*UPK3BL1*, *LCN2*, *S100A2*). In addition, we found that MP4 and MP5 accounted for a relatively high proportion, at 41.9% and 42.5% respectively in BC (**Figure 3E**) and MP5 accounted for a relatively high proportion, at 37.14% in TC (**Figure 3F**). We also concluded that MP2, MP4 and MP5 are key modules for BC and TC. Furthermore, we explored the functional enrichment of six modules. We observed that MP2 shows significant upregulation of pathways related to immune activation and stress response, including P53 signaling, IL2-STAT5 signaling, interferon-gamma response, and estrogen response. In MP4, estrogen response and bile acid metabolism are markedly upregulated. MP5 displays strong enrichment of estrogen response, PI3K-AKT-MTOR signaling and fatty acid metabolism (**Figure 3G**). To further delineate developmental trajectories, pseudotime analysis was conducted using the Slingshot algorithm. Stemness profiling via CytoTRACE revealed MP4-enriched malignant cells exhibited elevated stemness indices (**Figure 3H**). High-stemness cells were defined as trajectory origins, forming hierarchical branches in pseudotemporal ordering (**Figures 3I, J**). This indicates a potential transformation relationship among the co-pathogenic modules and suggests that these shared modules may drive malignant evolution in both BC and TC.

### Identification of key functional modules demonstrating the strongest positive association with disease progression using WGCNA

**Figure 4:**
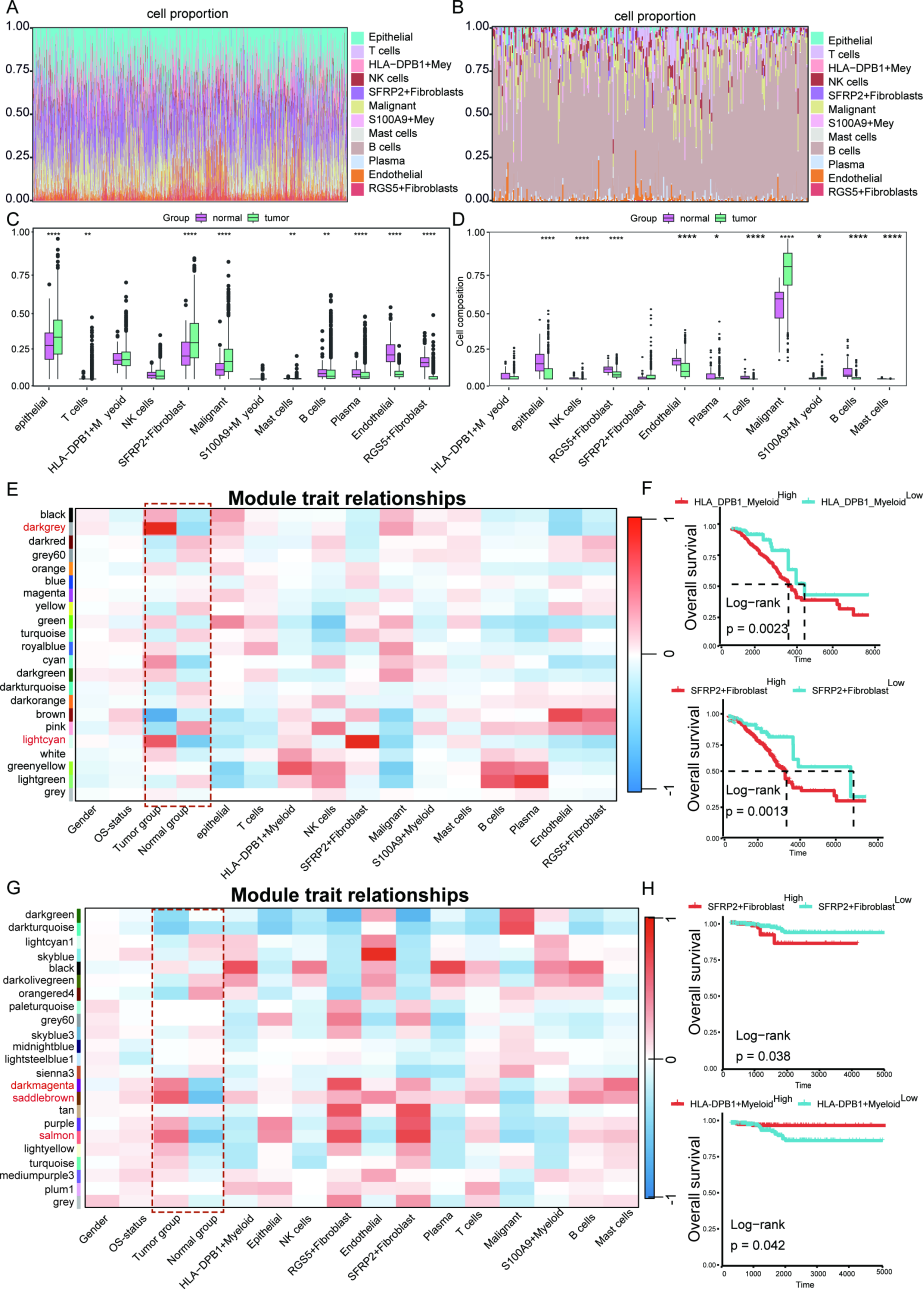
Identification of key functional modules demonstrating the strongest positive association with disease progression using WGCNA. ****p < 0.0001, **p < 0.01, *p < 0.05. **(A)** Reverse convolution analysis of BC scRNA-seq data via the “CIBERSORTx” algorithm to construct the feature matrix. **(B)** Reverse convolution analysis of TC scRNA-seq data via the “CIBERSORTx” algorithm to construct the feature matrix. **(C)** Difference analysis of immune cell in BC. **(D)** Difference analysis of immune cell in TC. **(E)** Relationships between gene modules and traits in BC, with correlation coefficients and P values provided for each cell. **(F)** Survival analysis revealed that HLA_DPB1_Myeloid and SFRP2+Fibroblast are associated survival of BC patients. **(G)** Relationships between gene modules and traits in TC, with correlation coefficients and P values provided for each cell. **(H)** Survival analysis revealed that RGS5+Fibroblast and HLA_DPB1_Myeloid are associated survival of TC patients.

To systematically identify gene modules linked to disease progression, we applied WGCNA to both BC and TC cohorts. CIBERSORTx, a computational deconvolution framework, was employed to resolve cellular heterogeneity between BC and TC using single-cell transcriptomic references. For BC, the single-cell atlas served as a reference to deconvolute 12 cell-type proportions across molecular subtypes via Wilcoxon rank-sum testing (**Figure 4A**). Analogously, TC-specific deconvolution was performed using its single-cell dataset (**Figure 4B**). Comparative analysis revealed BC-specific enrichment of epithelial cells, T cells, SFRP2+ fibroblasts, malignant cells, and mast cells, contrasted by depletion of B cells, plasma cells, endothelial cells, and RGS5+ fibroblasts (**Figure 4C**). Conversely, TC exhibited elevated malignant cells and S100A9+ myeloid cells, with significant reductions in epithelial cells, NK cells, RGS5+ fibroblasts, endothelial cells, plasma cells, T cells, B cells, and mast cells (**Figure 4D**). To delineate BC-associated gene modules, WGCNA was conducted using topological overlap matrix (TOM)-based hierarchical clustering. This identified 22 modules in BC and 23 in TC, with modules exhibiting *p* < 0.05 defined as clinically significant. In BC, the darkgrey and lightcyan modules showed the strongest positive correlation, encompassing 1,621 genes implicated in extracellular matrix remodeling (**Figure 4E**). Survival analysis revealed significantly reduced OS in high-infiltrating HLA-DPB1+Myeloid and SFRP2+Fibroblast subgroups (**Figure 4F**). Conversely, TC analysis identified darkmagenta, saddlebrown, and salmon as synergistically correlated modules, comprising 4,360 metabolism-related genes (**Figure 4G**). Strikingly, high SFRP2+Fibroblast infiltration predicted poor prognosis, while low HLA-DPB1+Myeloid infiltration was associated with adverse outcomes (**Figure 4H**). These findings indicate that SFRP2^+^ fibroblasts may represent a common key cellular population that regulates the TME and influences prognosis in both cancer types.

### Tumor microenvironment and cell communication

**Figure 5:**
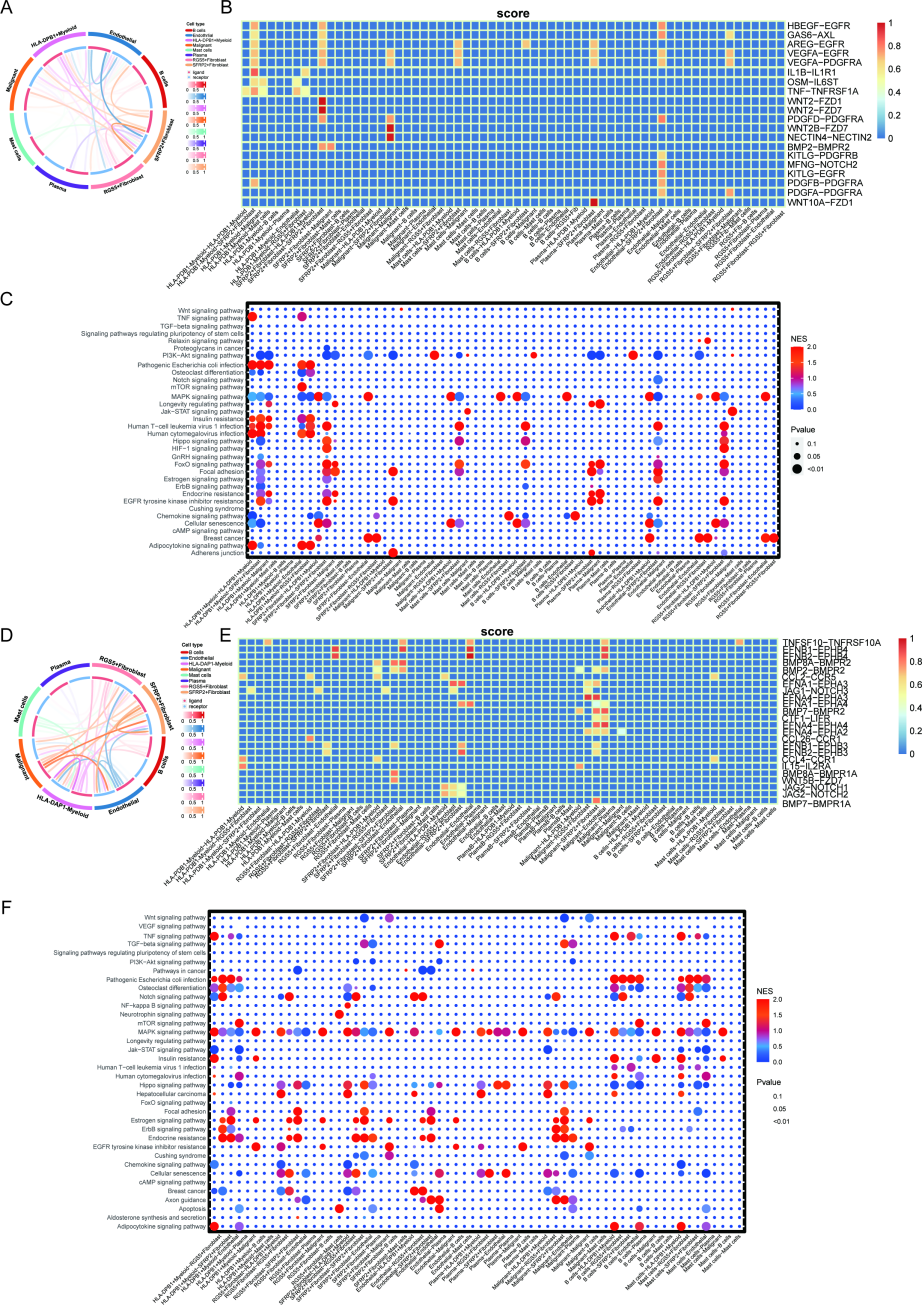
Tumor microenvironment and cell communication. **(A)** Cell-cell communication dynamics were mapped by integrating ligand-receptor co-expression patterns with Bayesian inference algorithms to quantify interaction likelihoods in BC. **(B)** The analysis of the receptor-ligand interaction in BC. **(C)** The analysis of the target gene pathway in BC. **(D)** Cell-cell communication dynamics were mapped by integrating ligand-receptor co-expression patterns with Bayesian inference algorithms to quantify interaction likelihoods in TC. **(E)** The analysis of the receptor-ligand interaction in TC. **(F)** The analysis of the target gene pathway in TC.

The TME constitutes a dynamic ecosystem wherein intercellular communication networks orchestrate oncogenic progression. Based on the above findings, we further utilized CellCall analysis to reveal the intercellular communication network. By integrating transcriptomic co-expression modules with ligand-receptor interactome profiling, we mapped context-specific signaling axes. Cellcall analysis revealed SFRP2+ fibroblasts as dominant communicators in BC, exhibiting elevated interaction strengths (**Figure 5A**). Ligand-receptor pair analysis identified WNT2B-FZD7 and NECTIN4-NECTIN2 as primary mediators of SFRP2+ fibroblast-malignant cell crosstalk in BC (**Figure 5B**). Downstream pathway enrichment demonstrated predominant activation of PI3K-AKT signaling, focal adhesion, EGFR tyrosine kinase inhibitor resistance, and adherens junction pathways (**Figure 5C**). In TC, SFRP2+ fibroblasts similarly demonstrated heightened communication centrality (**Figure 5D**), with distinct ligand-receptor pairs including *EFNA4*-*EPHA3*, *EFNA1*-*EPHA3*, *JAG1*-*NOTCH3*, and *EFNA4*-*EPHA2* (**Figure 5E**). Functional annotation uncovered enrichment of Wnt/β-catenin signaling, MAPK signaling pathway, EGFR inhibitor resistance, and estrogen response pathways (**Figure 5F**). These findings further confirm that SFRP2^+^ fibroblasts play a core regulatory role in the TME of both cancer types through distinct signaling axes.

### Construction and validation of an integrin-related signature via machine learning algorithms

**Figure 6:**
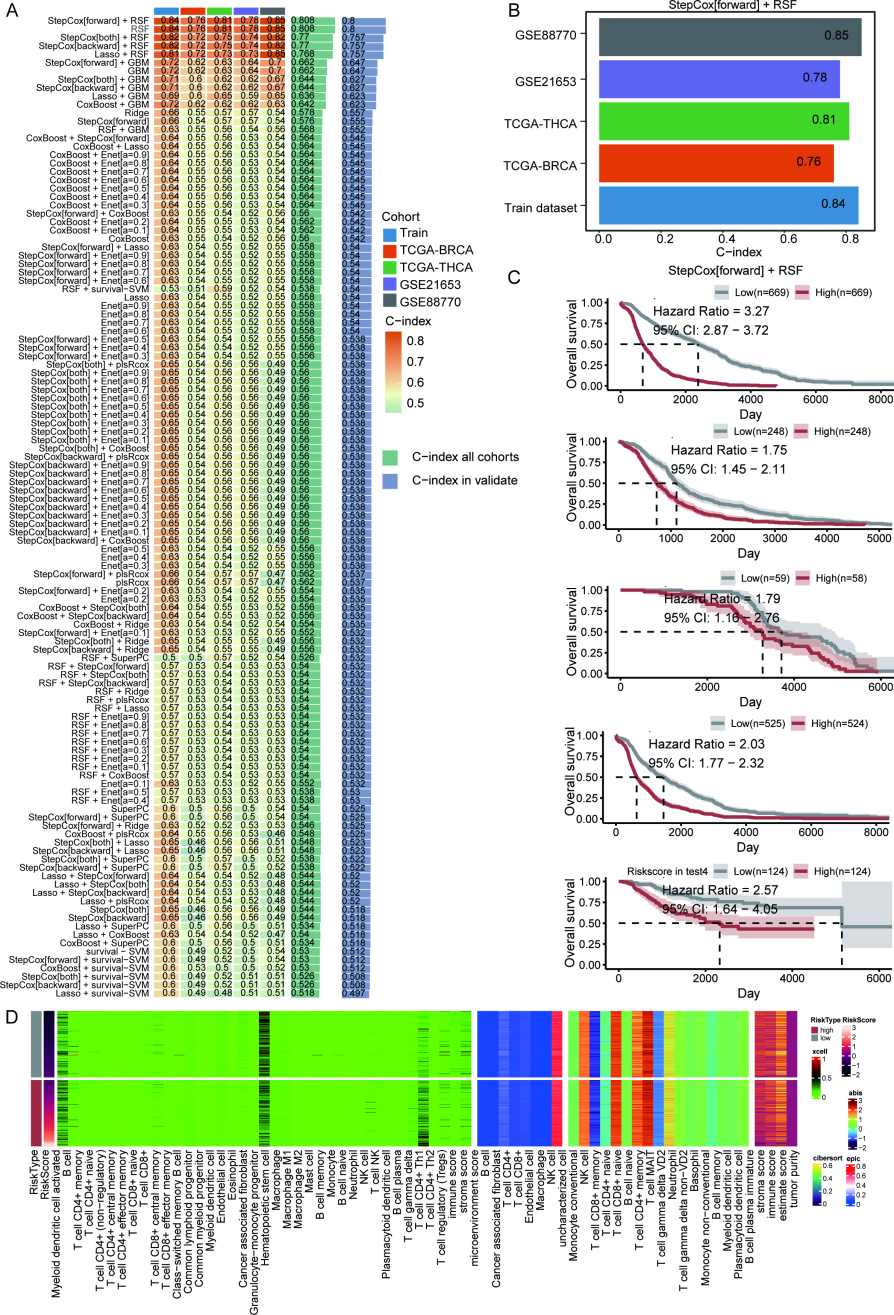
Construction and validation of an integrin-related signature via machine learning algorithms. **(A)** A total of 101 prediction models were developed across a tenfold cross-validation framework, and the C-index for each model was calculated for all the validation datasets. **(B)** The C-index for RSF. **(C)** Survival curves based on the risk model constructed with the training dataset, TCGA cohort, GSE88770 cohort, and GSE21653 cohort. **(D)** Immune cell analysis based on riskscore.

To develop a cross-cancer prognostic model, we systematically evaluated 101 machine learning algorithms. The top 12 models (ranked by mean concordance C-index) consistently incorporated GBM, StepCox, RSF, or hybrid parameterizations thereof. Notably, most of models demonstrated AUC > 0.76 in ROC analysis, with the top three exhibiting mean C-indices of 0.8 (**Figures 6A, B**). The backward elimination Cox model was prioritized for validation due to its parsimony and stability. Multi-cohort survival analysis confirmed significantly reduced overall survival in high-risk versus low-risk patients (**Figure 6C**). Comparative immune deconvolution across six algorithms revealed pronounced immunosuppression in high-risk tumors (**Figure 6D**). These results collectively establish the robust prognostic performance of our model and underscore its potential utility in guiding patient stratification for targeted immunotherapy.

### The SMR3B gene may act as a common pathogenic factor in both BC and TC

**Figure 7:**
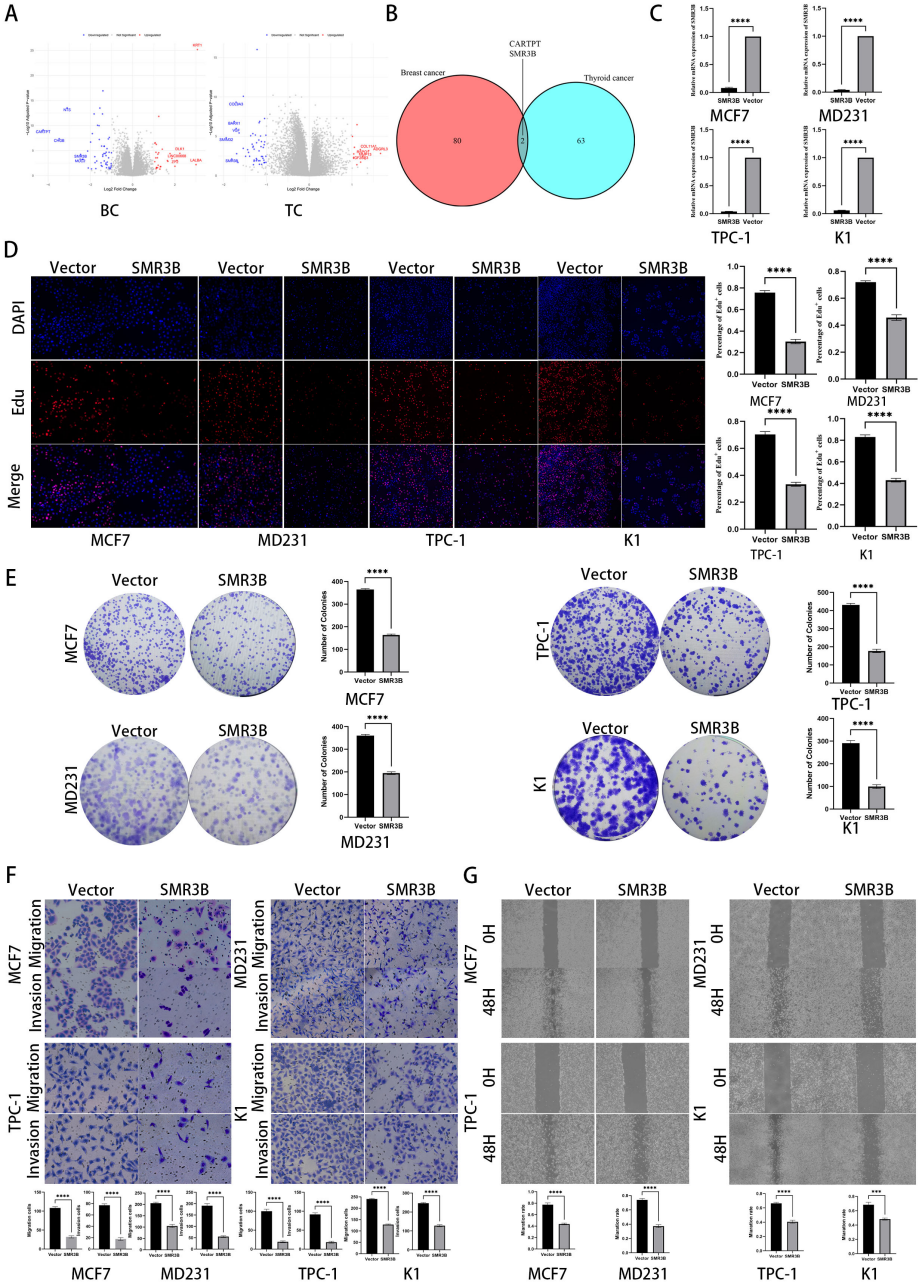
The SMR3B gene may act as a common pathogenic factor in both BC and TC. ***p < 0.001. **(A)** differential expression analysis based on high and low riskscore in BC and TC. **(B)** VENN plot displayed common DEGs in BC and TC. **(C)** qPCR verified SMR3B expression in BC and TC cell lines. **(D)** EDU assay revealed that SMR3B disturbed cell proliferation in BC and TC cell lines. **(E)** Colony formation assay revealed that SMR3B disturbed cell proliferation in BC and TC cell lines. **(F)** Transwell assay revealed that SMR3B disturbed cell migration and invasion in BC and TC cell lines. **(G)** Scratch assay revealed that SMR3B disturbed cell migration in BC and TC cell lines. **** P < 0.0001.

Based on the risk-stratification derived from the prognostic model, differential expression analysis was performed between high- and low-risk groups to identify key genes underlying the observed prognostic disparities. Differential expression analysis between high- and low-risk groups identified 82 breast cancer BC-specific and 65 TC-specific DEGs (adjusted *p* < 0.05, |log2FC|>1.0; **Figure 7A**), with CARPTP and SMR3B emerging as common DEGs (**Figure 7B**). SMR3B expression were detected in MCF7, MD231, TPC-1, and K1 cell lines in the two group (**Figure 7C**). Through EDU assay, we observed that disturbing SMR3B expression affects BC and TC cells proliferation ability (**Figure 7D**). Through colony formation assay, we discovered that disturbing SMR3B inhibited BC and TC cells colony formation ability (**Figure 7E**). Through Transwell assays, we concluded that disturbing SMR3B inhibited BC and TC cells migration and invasion ability (**Figure 7F**). Through scratch assay, we found that disturbing SMR3B inhibited BC and TC cells migration ability (**Figure 7G**). In conclusion, our experimental results collectively establish SMR3B as a prognostic biomarker in both breast cancer and thyroid cancer, significantly restraining key oncogenic phenotypes including proliferation, migration, and invasion of cancer cells.

## Discussion

BC and TC represent two prevalent malignancies in women, frequently exhibiting synchronous occurrence (10). Some studies have found that the risk of TC in first-degree relatives of BC patients is significantly increased. However, earlier studies have suggested from a pathological perspective that thyroid dysfunction, dysregulation of estrogen signaling pathways, and common lifestyle/environmental exposure may be independent risk factors for BC and TC (30, 31). These findings highlight a possible link between shared genetic factors and overlapping hormonal pathways in BC and TC co-development; however, the exact molecular mechanisms connecting these factors to their joint pathogenesis remain unclear.

Our study conducted a systematic multi-omics investigation into the pathogenic interplay between BC and TC, uncovering their shared molecular landscape. Integrated multi-omics analyses identified conserved DEGs, with KEGG enrichment highlighting the JAK-STAT signaling pathway and cytokine-cytokine receptor interactions as central co-pathogenic mechanisms. These pathways emerged as core drivers of oncogenic convergence in both malignancies. Notably, Limberg et al. demonstrated that JAK/STAT pathway activation mediates BRAFV600E inhibitor (BRAFi) resistance in TC, and combinatorial targeting of BRAFi with JAK/STAT inhibitors suppressed BRAFV600E+ TC cell proliferation (32). Prior investigations have demonstrated that genetic or pharmacological inactivation of the JAK-STAT signaling pathway significantly suppresses TC progression (33, 34). Meanwhile, previous studies concluded that JAK/STAT pathway involved in BC progression. CXCL9 reshaped triple-negative BC TME by activating JAK/STAT pathway (35). TRIM22 attenuates BC progression by targeting the JAK-STAT/STAT3 axis through CCS-mediated degradation, resulting in suppressed STAT3 phosphorylation and reduced tumor invasiveness (36). CircNOL10 inhibits mammary tumorigenesis via miR-767-5p-dependent SOCS2 upregulation, which blocks JAK2/STAT5 signaling, thereby impairing tumor growth and metastatic dissemination (37). Furthermore, cytokine-cytokine receptor crosstalk mediates oleic acid (OA)-induced IL-10 secretion in BC, mechanistically linking Lactobacillus-enriched gut microbiota to antitumor immunity (38).

Moreover, the scRNA-seq analysis further revealed the unique and overlapping characteristics of the TME and demonstrated key signaling nodes driving disease progression in both conditions These findings not only elucidate the mechanistic links between BC and TC pathogenesis but also reveal novel therapeutic strategies for targeting their shared disease pathways. Our study also underscores the intra-tumoral heterogeneity of malignant cells, with NMF revealing distinct gene expression programs within BC and TC. Notably, several of these programs are associated with p53 signaling, apoptosis, TGF-beta signaling and hypoxia, emphasizing the critical role of the microenvironment in disease progression.

Moreover, scRNA-seq analysis delineated both unique and convergent TME features between BC and TC, identifying core signaling hubs that orchestrate cross-cancer progression. These insights not only establish mechanistic convergence between BC and TC pathogenesis but also reveal actionable therapeutic targets within their shared pathways. NMF further resolved intra-tumoral heterogeneity in malignant cells, uncovering six functionally distinct transcriptional programs. Critical programs were mechanistically linked to p53 signaling, apoptosis, TGF-beta signaling and hypoxia, collectively underscoring microenvironmental crosstalk as a linchpin of metastatic evolution.

WGCNA identified critical gene modules significantly correlated with disease states, underscoring the pivotal role of epithelial cell and fibroblasts in shaping the microenvironment of BC and TC. These findings deepen the understanding of the tumor microenvironment and its potential contribution to disease progression. In addition, cell communication analysis revealed that SFRP2+ Fibroblast might be a key component in reshaping the comorbidity immune microenvironment. Montagner, M. et al. concluded that SFRP2+ fibroblasts promote BC dormancy via fibronectin-integrin survival signaling, targeting SFRP2 reduces metastatic burden (39). SFRP2+ fibroblasts epigenetically drive DCIS-to-IDC progression by downregulating expression to activate pro-invasive signaling pathways and remodel the immunosuppressive microenvironment, enabling malignant transition (40). To systematically explore shared molecular drivers, we developed a prognostic model based on an advanced machine learning framework incorporating 101 algorithm combinations across 10 distinct approaches. Among the identified candidates, submaxillary gland androgen regulated protein 3B (SMR3B) emerged as a top prognostic gene, showing consistently downregulation in both BC and TC.

Emerging multi-disease evidence implicates SMR3B as a pleiotropic modulator of inflammatory and neoplastic processes. In periodontal inflammation (PA) research, SMR3B expression was notably upregulated in the salivary proteome of PA patients and their offspring compared to healthy controls, suggesting its potential involvement in inflammatory processes or tissue repair mechanisms associated with PA pathogenesis (41). The consistent absence of SMR3B—a salivary gland-specific protein—and its associated peptides in both tumor tissue extracts and patient saliva samples highlights its potential as a hallmark feature of salivary gland tumors, suggesting tumor-induced suppression of gland-specific protein expression (42). SMR3B, identified as a core component of a prognostic model, contributes to a multi-gene prognostic model that effectively stratifies recurrence risk and guides personalized chemotherapy decisions in BC patients (43, 44). In the study by Giri, K. et al., SMR3B was identified as a salivary biomarker whose specific peptides (GPYP/IPPP), combined with other protein markers, effectively distinguished triple-negative breast cancer patients (80% sensitivity, 95% specificity), though its precise biological mechanisms remain unclear (45). Despite limited studies in thyroid cancer, SMR3B’s endocrine/immune-metabolic roles suggest biomarker potential, warranting further exploration. These findings collectively position SMR3B as a novel pan-cancer therapeutic target bridging inflammatory remodeling and neoplastic transformation.

In conclusion, this study systematically demonstrates the pathological convergence between BC and TC, underscoring the necessity of an integrated approach to unravel their complex interplay. Our findings reveal that shared molecular pathways such as JAK-STAT signaling pathway is a central driver of disease progression in both BC and TC. Besides, SMR3B identified in this study and other core mediators may present new opportunities for therapeutic intervention for dual efficacy. Although our single-cell transcriptomic analysis is comprehensive, it is constrained by dataset availability, which may limit its ability to fully capture patient heterogeneity across different disease stages. By elucidating the molecular crosstalk between these conditions, our study offers novel insights into their interconnected mechanisms and enables precision medicine strategies for these comorbid conditions.

In conclusion, this study unveils convergent molecular mechanisms underlying breast and thyroid cancers, emphasizing the pivotal role of JAK-STAT signaling and cytokine networks in their co-pathogenesis. Single-cell and multi-omics integration revealed shared microenvironmental dynamics, particularly SFRP2+ fibroblast-mediated crosstalk driving oncogenic plasticity. We identified key functional gene modules linked to tumor stemness and therapy resistance, while machine learning prioritized SMR3B as a prognostic biomarker attenuating proliferation, invasion, and metastasis. These findings establish a framework for dual-targeted therapeutic strategies and underscore the necessity of cross-disease molecular profiling to address endocrine-related cancer comorbidities.

## Data Availability

Publicly available datasets were analyzed in this study. This data can be found here: GSE228499, GSE232237, GSE21653, GSE88770 cohorts were retrieved from GEO (https://www.ncbi.nlm.nih.gov/geo/) and TCGA-BRCA, TCGA-THCA cohorts (https://portal.gdc.cancer.gov).
